# The role of fermented foods in managing food allergies in children and adults: a systematic review

**DOI:** 10.3389/fnut.2025.1689636

**Published:** 2026-01-05

**Authors:** Bahtir Hyseni, Konstantinos Papadimitriou, Aline Issa, Ayşe Nur Tonay, Burcu Gündüz Ergün, Carmen Maria Gonzalez-Domenech, Elena Arranz, Endra Luzha Pula, Erenay Erem, Enriqueta Garcia-Gutierrez, Gregory Bouchaud, Hania Szajewska, Hatice Kalkan Yıldırım, İbrahim Ender Künili, Lidia Markiewicz, Mario Caruana Grech Perry, Meral Kilic-Akyilmaz, Mounaim Halim El Jalil, Ryma Merabti, Sandra Mojsova, Yonca Karagül Yüceer, Zehra Gulsunoglu-Konuskan, Aslı Akpınar, Barçın Karakaş-Budak, Christophe Chassard, Smilja Pracer, Guy Vergères, Simona Lucia Bavaro

**Affiliations:** 1 Faculty of Food Technology, University “Isa Boletini” in Mitrovica, Mitrovica, Republic of Kosovo; 2 Laboratory of Food Quality Control and Hygiene, Department of Food Science and Human Nutrition, Agricultural University of Athens, Athens, Greece; 3 Faculty of Nursing and Health Sciences, Notre Dame University- Louaize, Zouk Mosbeh, Lebanon; 4 UCD School of Biosystems and Food Engineering, University College Dublin, Dublin, Ireland; 5 Department of Molecular Biology and Genetics, Yıldız Technical University, İstanbul, Türkiye; 6 Department of Microbiology, School of Medicine, University of Malaga, Málaga, Spain; 7 Universidad Autónoma de Madrid (CEI UAM+CSIC) and Institute of Food Science Research (CIAL, CSIC-UAM), Madrid, Spain; 8 Food Hygiene and Technology Department, Faculty of Veterinary Medicine, Istanbul University-Cerrahpaşa, İstanbul, Türkiye; 9 Department of Food Engineering, Faculty of Chemical Metallurgical Engineering, Istanbul Technical University, İstanbul, Türkiye; 10 Agronomic Engineering Department, Technical University of Cartagena, Cartagena, Spain; 11 INRAE, UR 1268 BIA, Nantes, France; 12 Department of Paediatrics, The Medical University of Warsaw, Warszawa, Poland; 13 Department of Food Engineering, Faculty of Engineering, Ege University, Izmir, Türkiye; 14 Faculty of Marine Sciences and Technology, Canakkale Onsekiz Mart University, Çanakkale, Türkiye; 15 Immunology and Food Microbiology Group, Institute of Animal Reproduction and Food Research, Polish Academy of Sciences, Olsztyn, Poland; 16 Department of Food Science, Nutrition & Dietetics, Faculty of Health Sciences, University of Malta, Msida, Malta; 17 Higher School of Technology, Mohammed V University of Rabat, Rabat, Morocco; 18 Department of Cellular and Molecular Biology, Abbes Laghrour University, Khenchela, Algeria; 19 Laboratory of Biotechnology and Food Quality, Institute of Nutrition, Food and Agri-Food Technologies, Constantine 1 University, Constantine, Algeria; 20 Department of Food Safety and Veterinary Public Health, National Veterinary and Food Institute, Faculty of Veterinary Medicine, University Ss Cyril and Methodius, Skopje, North Macedonia; 21 Department of Food Engineering, Çanakkale Onsekiz Mart University, Çanakkale, Türkiye; 22 Faculty of Health Sciences, Nutrition and Dietetics Department, Istanbul Aydin University, Istanbul, Türkiye; 23 Department of Food Engineering, Manisa Celal Bayar University Faculty of Engineering and Natural Science, Manisa, Türkiye; 24 Department of Food Engineering, Akdeniz University Faculty of Engineering, Antalya, Türkiye; 25 UCA, INRAE, VetAgro Sup, UMRF 0545, 15000, Aurillac, France; 26 Institute for Biological Research Siniša Stanković, National Institute of the Republic of Serbia, University of Belgrade, Belgrade, Serbia; 27 Agroscope, Bern, Switzerland; 28 Institute of Sciences of Food Production (ISPA)-National Research Council (CNR), Grugliasco, Italy

**Keywords:** fermented foods, food allergy, immunomodulation, gut microbiota, lactic acid bacteria, protein hydrolysis, systematic review, hypoallergenicity

## Abstract

**Introduction:**

Fermented foods are among the oldest foods produced, and several different health benefits are attributed to their consumption even in the absence of concrete clinical evidence. To address this gap, this systematic review focuses on the effects of the consumption of fermented foods on food allergies.

**Methods:**

This systematic review was conducted following the Preferred Reporting Items for Systematic Reviews and Meta-Analyses and the relevant European Food Safety Authority guidelines. A systematic search strategy was established and registered in a study protocol in Open Science Framework. Scopus, MEDLINE, and Cochrane Library were searched with specific strings targeting human studies focusing on Fermented food and food allergies. Inclusion and exclusion criteria were defined based on the People Intervention Comparison Outcome elements. The Cadima tool was used to perform screening and selection of articles. A standard template was used for data extraction. Risk of bias assessment was performed using the Risk of Bias 2.0 Tool, Risk of Bias in Non-randomized Studies - of Interventions, or Newcastle–Ottawa Scale protocols. Additionally, a narrative section was written based on the European Food Safety Authority guidelines for the mechanism of action and product characteristics for evidence support.

**Results:**

From a total of 558 initial records, 10 studies were finally selected. Fermented foods evaluated were fermented soy products, baked goods, fruit-based beverages, vinegar-treated foods, oat-based drinks, and dairy products (yogurt, cheese). In several studies, a reduced allergenicity was reported that was related to fermentation-mediated hydrolysis of allergenic proteins of gluten or soy. Additional mechanisms were related to anti-allergic immunomodulatory effects or favorable shifts in gut microbiota composition. In one case, fermented food consumption led to aggravation of the allergic response, presumably due to the compounds generated during soy fermentation. Risk of bias assessment revealed that most studies were performed with important methodological limitations.

**Conclusion:**

While fermented foods hold promise in reducing food allergenicity and promoting tolerance, current evidence is limited to draw solid conclusions. Rigorous, well-designed human clinical trials, complemented by mechanistic studies *in vitro* and *in vivo*, are needed to clarify the role of fermented foods as dietary or even clinical tools to combat food allergies.

**Systematic review registration:**

https://osf.io/hgjaf/10.17605/OSF.IO/HGJAF.

## 1 Introduction

Fermentation, one of the oldest food preservation techniques, yields a diverse array of fermented foods (FFs) that have been part of the human diet for centuries. FFs can be classified based on their plant or animal origin, including those made from cereals, vegetables, legumes, roots and tubers, milk, meat, fish, and other miscellaneous sources. They range from traditional staples such as yogurt, kefir, bread, fermented meat, and pickled vegetables to more recent innovative foods like novel plant-based analogues of fermented dairy and meat (1–4). FFs are the products of the metabolic activity of fermentative microorganisms such as bacteria, yeast, or molds that can convert sugars and other molecules into alcohol, acids, and flavor compounds in order to preserve food and develop desirable organoleptic characteristics (3, 5, 6). Under favorable conditions, fermenting microbes dominate the microbial ecosystem of FFs. Their metabolic activity simultaneously inhibits spoilage and pathogenic microbes through competition, pH reduction, alcohol production, and the release of many different antimicrobial compounds in the food matrix. Furthermore, fermentation enhances nutrient bioavailability and reduces anti-nutritional compounds, contributing to a healthy diet (7–9). Multi-omics approaches are rapidly advancing our understanding of the diverse bioactive compounds produced during the fermentation of plant and animal raw materials, enabling evaluation of the composition and potential health effects of FFs (10, 11). Depending on the specific technological process, fermentation can result directly in the final product or serve as a step in the production of other foods (e.g., sourdough bread). The long history of safe use of FFs is accompanied by a broad notion that they are beneficial for consumers’ health in several different aspects; however, solid scientific evidence for such properties is often missing.

Food allergies refer to immune-mediated hypersensitivity reactions that can be triggered by different dietary components, leading to symptoms ranging from mild gastrointestinal discomfort to life-threatening anaphylaxis. Beyond the nutritional value and diversity of FFs, the fermentation process *per se* shows promise in mitigating food allergies (12). Immunomodulatory mechanisms that reduce allergic responses generally involve the reduction of hypersensitivity by promoting immune tolerance, suppressing inflammation, and restoring immune signaling pathways. Fermentation may contribute to this by modifying the structure and composition of allergenic proteins, shifting the Th1/Th2 balance towards a Th1 response, increasing the secretion of regulatory cytokines, and reducing allergy-related markers (12, 13). Fermenting microorganisms act as natural “digestive agents,” breaking down allergenic proteins before gastrointestinal digestion, thus mitigating the formation of immune-triggering peptides (14–16). For example, it has been demonstrated that many microbial starter cultures, like lactic acid bacteria (LAB), yeasts, and molds, have the ability to degrade soy proteins into smaller peptides during fermentation, reducing their allergenicity (14, 16–18). While promising results have been observed in a variety of FFs, the actual impact of fermentation on food allergenicity is complex. Indeed, in certain meat products, fermentation can paradoxically increase allergenicity, particularly when certain starters, such as *Penicillium* sp., are used (19–21). Beyond their impact on allergenic proteins, FFs provide valuable live microorganisms with probiotic potential, which may have an impact on food allergies after consumption (22). For example, *Lactobacillus* starter strains have been shown to enhance innate and specific immune responses, potentially improving children’s allergy-related immune parameters by modulating immunoglobulin E (IgE) levels and regulatory T cell activity (23–25). In addition, gut dysbiosis, an imbalance of the gut microbiome, is a known risk factor for immune-mediated conditions, including food allergies (26). Certain microbial starters have the potential to promote gut eubiosis and foster a healthy microbiota, which could in turn mitigate the development of food allergies (27–29).

In this light, food allergies have a profound impact on the quality of life of affected individuals and their families, as well as on healthcare costs and food system management. Understanding the extent to which specific fermented foods may alleviate or exacerbate allergic reactions is therefore of high societal relevance. Such knowledge can help identify safer dietary options for everyday consumption, enable clinicians to offer more evidence-based dietary guidance, and support policymakers and the food industry in promoting clearer labeling and greater transparency for consumers.

Despite the growing interest in the health benefits of FFs, their relevance to food allergy remains largely unexplored due to the limited availability of robust human clinical evidence. To the best of our knowledge, the European Academy of Allergy and Clinical Immunology (EAACI), for example, does not provide specific recommendations regarding fermented foods. The present systematic review addresses this gap by systematically evaluating the available human studies on the effects of fermented food consumption in the prevention and management of food allergies, focusing on their impact in both allergic individuals and populations at high risk of developing food allergies. This review also highlights the need for additional high-quality studies to further explore the role of FFs in the prevention and management of food allergies.

## 2 Methods

### 2.1 Methodology

This systematic review was conducted following the protocol of the Preferred Reporting Items for Systematic Reviews and Meta-Analyses (PRISMA) (30), the guidance of the European Food Safety Authority (EFSA) (31), and the guidelines reported in the study by Muka et al. (32). The selection of articles, eligibility assessment, data extraction, and statistical analysis were performed in accordance with a predefined study protocol. This review has been conducted within the framework of Working Group 3 of the PIMENTO project, which is part of a COST Action initiative CA20128 focused on advancing research and innovation in the area of fermented foods.

### 2.2 Search strategy

In this systematic review, relevant studies were identified using a structured and comprehensive search strategy. Scopus, MEDLINE, and The Cochrane Library were searched for articles published from January 1, 1970, to December 31, 2024. A generic search string was developed for the PIMENTO project, which included fermented foods, while review-specific search strings were used for food allergies, IgE-mediated allergies, non-IgE-mediated allergies, and food allergy diagnostic tests. Only English-language publications were considered eligible, and only peer-reviewed journal articles were included, with gray literature explicitly excluded. No restrictions were placed on the geographic region or study design (e.g., observational or interventional human studies). The systematic review was registered in the Open Science Framework (OSF) and is cited with its unique identifier and DOI link (doi: 10.17605/OSF.IO/HGJAF). The complete search string is documented as a Supplementary file to ensure transparency and reproducibility (Supplementary Table 1).

### 2.3 Inclusion and exclusion criteria

The inclusion and exclusion criteria were defined based on the Population, Intervention, Exposure, and Outcome (PI(E)O) elements: Participants, Intervention/Exposure, and Outcomes (32). The quality of the negative control for FFs tested was assessed for all studies meeting the PI(E)O criteria. Conference proceedings, abstracts, and non-peer-reviewed literature were excluded. The selection process adhered strictly to the following inclusion and exclusion criteria:

Inclusion criteria:

1. Human intervention and observational studies focusing on children and adults (6 months–65 years old) with food allergies, including IgE-mediated, non-IgE-mediated, or mixed mechanisms. Studying both children and adults is essential because immune system development and dietary patterns differ significantly across these age groups.
2. Studies evaluating health-related outcomes such as allergic symptoms, growth parameters, and the prevalence of food allergies following fermented food consumption.
3. Studies confirming food allergies through double-blind placebo-controlled food challenges (DBPCFC), skin prick tests (SPT), or other validated diagnostic tools.
4. Studies concerning individuals at high risk of food allergies due to personal history of atopy or a first-degree relative (e.g., parent or sibling) with an atopic condition (e.g., asthma, allergic rhinitis, food allergy, or eczema).

Exclusion criteria:

1. Non-human or *in vitro* studies.
2. Studies conducted on pregnant women, infants under 6 months, or adults over 65 years.
3. Studies addressing lactose intolerance, FODMAP intolerance, or non-celiac, non-allergic gluten sensitivity.
4. Studies on alcoholic beverages with an alcohol content exceeding 1.25%.

### 2.4 Study selection

The Cadima tool was employed for the screening and selection process of scientific articles (33). Two independent reviewers conducted this process in three phases, following consistency checks to ensure adherence to the study protocol standards. In the first phase, articles were initially evaluated based on their titles and abstracts. Duplicates and irrelevant studies were excluded to streamline the selection process and ensure efficiency. Subsequently, articles that passed the initial screening were subjected to a thorough full-text review to determine their eligibility. Cadima software played a critical role in systematically tracking and documenting decisions, which ensured adherence to the study protocol. Finally, all the articles selected during the second phase were checked by two additional co-authors whenever consensus was necessary. In addition to the primary selection process, supplementary sources were also considered. One potentially relevant systematic review was identified (34); however, after further inspection, references therein were found not to be relevant to this study.

### 2.5 Data extraction

Data extraction followed a clear and consistent approach that matched the review objectives. An Excel template was used for data extraction, prepared for the PIMENTO-DE framework (Supplementary Table 1). Two independent reviewers performed data extraction. Disagreements between two reviewers were resolved through consensus or consultation with a third reviewer.

### 2.6 Risk of bias assessment

The studies selected for the systematic part of the review were further analyzed for risk of bias concerning their design. Two reviewers were assigned to each study. Differences were settled after discussion between the two reviewers or after the intervention of a third reviewer. The quality of randomized interventional studies was assessed using the Risk of Bias 2.0 (ROB 2.0) tool (35), the non-randomized interventional studies were assessed using the Risk of Bias In Non-randomized Studies of Interventions (ROBINS-I) tool (36), and the observational studies were assessed using the Newcastle–Ottawa Scale (NOS)^1^.

### 2.7 Methodology of non-systematic part

Following the EFSA requirements, a narrative part on additional *in vitro* experimental evidence and animal tests were presented in order to provide more information related to the outcomes of the human studies described in this study. Relevant experiments and evidence were discussed, focusing on the key mechanisms that might affect the interactions between the FFs and the immunological reaction occurring in food allergy, their bioavailability, and product characteristics.

## 3 Results and discussion

### 3.1 PRISMA diagram

**Figure 1:**
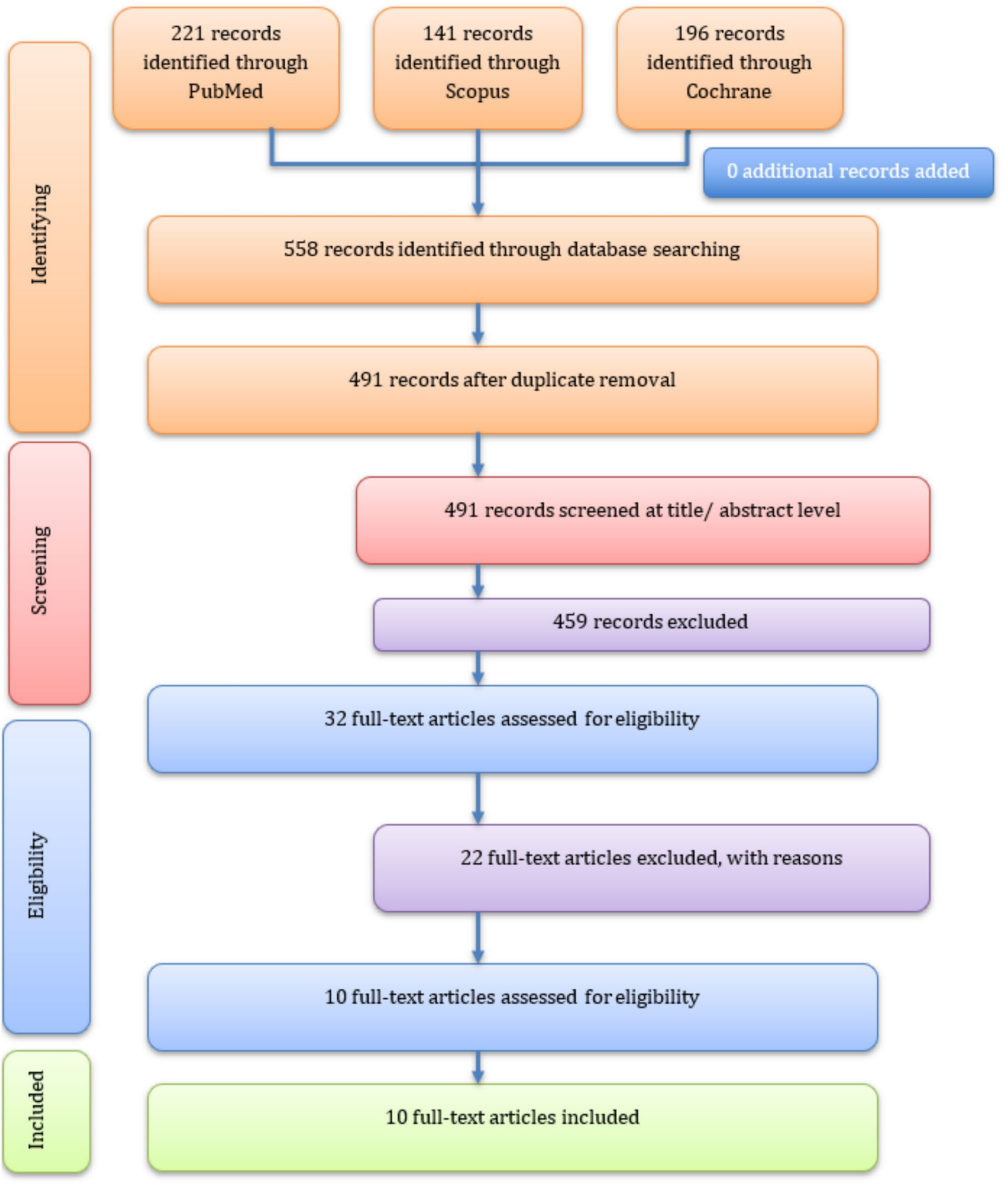
PRISMA diagram summary of the process of the literature search.

The literature search was conducted according to the Population, Intervention, Comparison, and Outcomes (PICO) criteria (32). In total, 558 papers were retrieved from three databases using the search strategy presented in Supplementary Table 1. After removal of duplicate entries, 491 papers were screened based on their title and abstract for eligibility (Figure 1). A total of 459 articles were excluded based on the first screening, leaving 32 articles for full-text screening. Eight studies were found not to be related to FFs; five did not concern a food allergy, four had no full-text article published, two were review papers, one was an animal study, one focused on an alcoholic beverage, and one was only a study protocol. Ten studies were retained for final data extraction and systematic review.

### 3.2 The fermented foods matrices

In the majority of the studies selected for this systematic review, the FFs explored to assess their potential effect on food allergies were of plant origin, including soy derivatives, cereal-based foods, and products from fruit fermentations (Table 1). Only two studies concerned fermented dairy products, specifically cheese and yogurt.

**Table 1:** Food matrices employed in the studies collected for the assessment of FFs on food allergies.

Food groups	References	Fermented food	Microorganisms	Raw material/ substrates
Soy products (meals, sauce)	(37)	Soy sauce “shoyu”	not mentioned	Soybean and wheat
Wheat, barley, millet, oat, lentils, buckwheat, soybeans products (breads, cakes, biscuits)	(38)	Hydrolyzed wheat baked goods (biscuits and cakes)	*F. sanfranciscensis* *Lat. alimentarius* *Lev. brevis* *Len. hilgardii*	Wheat (*Triticum aestivum* cv. Appulo) flour plus fungal proteases
	(39)	Sourdough bread	*F. sanfranciscensis* *Lat. alimentarius* *Lev. brevis* *Len. hilgardii*	Mix of wheat (*Triticum aestivum*), oat (*Avena sativa*), millet (*Panicum miliaceum*), and buckwheat (*Fagopyrum esculentum*) flours plus cell extracts
	(40)	Gluten-free sourdough wheat baked goods	*F. sanfranciscensis* *Lat. alimentarius* *Lev. brevis* *Len. Hilgardii*	Wheat (*Taestivum* cv. Appulo) flour plus fungal proteases
	(41)	Various bread types (soft white bread, rye bread, soft wholemeal bread, crisp bread, crispy flatbread, dishes based on bread)	not mentioned	Various grains (wheat, rye, etc.)
Fruit products (juices and vinegar)	(42)	Citrus juice	*Lac. plantarum* (heat killed)	Satsuma mandarin (*Citrus unshiu*)
	(43)	Vinegar (used for marination of egg, chicken and lentil)	Not mentioned	White wine produced from white grapes
Novel plant-based products (Sprouted and fermented food)	(44)	Sprouted oat fermented beverage	*Lac. plantarum*	Oat flour obtained from oat grains of Meeri variety
Milk products (cheese)	(45)	Semi-fat hard cheese (Grana Padano)	not mentioned	Milk
	(46)	Yogurt	not mentioned	Milk

Sugiura and Sugiura (37) investigated the properties of seven different fermented sauces. These sauces were mostly derived from soy (koikuchi soy sauces), while the rest were from fish, millet (kibi), broad bean, and barnyard. Cereal-based matrices included hydrolyzed wheat baked goods, sprouted-grain breads, and sourdough breads. In the study by Greco et al. (38), biscuits and cakes were produced using wheat flour hydrolyzed by LAB or LAB combined with fungal proteases. In the first case, certain strains formerly belonging to the Lactobacillus genus, namely of *Companilactobacillus alimentarius* (*Lactobacillus alimentarius*), *Levilactobacillus brevis, Fructilactobacillus sanfranciscensis,* and *Lentilactobacillus hilgardii* were employed for sourdough fermentation. In the second fermentation, six strains of *F. sanfranciscensis* exhibiting peptidase activity against proline-rich peptides were employed, while proteases from *Aspergillus oryzae* and *A. niger* was also added to further improve the properties of the dough. The LAB strains originated from Italian natural wheat sourdough were used for the production of bread. Furthermore, Di Cagno et al. (39) investigated the properties of sourdough bread made from wheat combined with non-toxic flours from oat, millet, and buckwheat. Bread samples were fermented either by yeast or lactobacilli. In the second case, cytoplasmic extracts of the LAB were also added to the dough. The LAB used as starters and for the production of cytoplasmic extracts were *C. alimentarius*, *Lev. brevis*, *F. sanfranciscensis*, and *Len. hilgardii*, which were preselected for their ability to hydrolyze gliadin fractions of wheat sourdoughs. In a later study, Di Cagno et al. (40) rendered sourdough made by wheat flour (cv. Appulo) gluten-free by extending the fermentation for 48 h and adding selected lactobacilli strains and proteases from *A. oryzae* and *A. niger*. In this case, they produced sweet baked goods. Furthermore, Segerstad et al. (41) recorded the dietary intake of gluten through the consumption of various bread types (soft white bread, rye bread, soft wholemeal bread, crisp bread, crispy flatbread, dishes based on bread) during the Environmental Determinants of Diabetes in the Young (TEDDY) multicenter study and Celiac Disease (CD) development.

Concerning FFs derived from fruit, Yamamoto-Hanada and colleagues (42) fermented Satsuma mandarin juice with *Lactiplantibacillus plantarum* (*Lactobacillus plantarum*) YIT 0132, a strain originally isolated from homemade pickles. After fermentation, the juice was heat-treated to kill the cells of the starter and convert it to a carrier of paraprobiotic components. Armentia et al. (43) explored the properties of vinegar from white wine to reduce the allergenicity of food extracts derived from the boiling of eggs, lentils, and chicken meat.

A novel FF was developed by Aparicio-Garcia et al. (44), named sprouted oat fermented beverage (SOFB). Oat grains were first germinated, freeze-dried, and milled into sprouted oat flour. After heat treatment to reduce microbial loads, the flour was mixed with water, sucralose, salt, and sodium bicarbonate. *L. plantarum* was used for the fermentation. The fermented beverage produced was validated to be gluten-free.

In the study by Marseglia et al. (45), two traditional Padano (PDO) Italian cheeses were investigated, i.e., Grana Padano and Trentin Grana. Both cheeses were produced with typical processes from the same batch of milk. The only difference was that in the first cheese, egg lysozyme was also added. Samples of both cheeses, ripened for 12 or 24 months, were tested. Finally, an observational study by Özmert et al. (46) described the role of regular yogurt consumption during the weaning period of children in Türkiye. No additional information is provided for the yogurt.

Overall, the FFs represent a wide range of substrates and microbial ecosystems that were investigated. In certain instances, fermenting LAB were specifically chosen to interact with potential allergenic substrates within the food matrix. Microbial enzymes or cell lysates were also added to the raw source or food matrix in an effort to further decrease the degree of allergenicity of the final product.

### 3.3 Description of the selected human studies

The objective of this systematic review is to comprehensively synthesize the available human evidence examining the relationship between fermented food consumption and allergic responses, to critically appraise the methodological quality of the included studies, and to contextualize the proposed mechanisms of action that may explain these effects, without overstating the level of certainty given the limited and heterogeneous evidence currently available.

To assess the effect of consumption of FFs on food allergies, data were systematically extracted from the 10 included studies (Tables 2, 3). The case report by Sugiura and Sugiura (37) in Japan documented soy sauce allergy in four female patients aged 10, 35, 46, and 51 who developed cellulitis and dermatitis around the lips by consuming meals with soy sauce. Despite negative specific IgE (sIgE) tests for soy and wheat, skin prick testing confirmed an allergic reaction to soy sauce, but not its individual components, such as soy, wheat, fish, millet, barnyard grass, and common additives, which tested negative. Histamine levels were reported to be high in darker soy sauces, up to 9 mg/100 g or in broad bean sauce up to 76 mg/100 g. However, histamine poisoning was eliminated as the cause, since nine volunteers experienced negative skin prick test results after testing with soy sauce. The allergy was attributed to unknown substances formed during the fermentation process.

**Table 2:** Characteristics of studies eligible for the systematic part.

Reference	Fermented food	Type of allergy	Study design	Population	Region
(37)	Soy sauce (shoyu)	Soy sauce allergy	Case report	Four female patients aged 10, 35, 46, 51	Japan
(38)	Baked goods sourdough fermentation with *Lactobacilli* and fungal proteases	Celiac disease	Clinical challenge study	16 celiac disease patients aged 12–23 years who were on a strict gluten-free diet for at least 5 years	
(39)	Sourdough bread wheat (30%), oat, millet, and buckwheat flours fermented with *Lactobacilli*	Celiac disease (gluten-sensitive enteropathy)	In vivo double-blind, acute challenge study	Twenty volunteer CS patients were recruited after at least 2 years on a gluten-free diet, negative test for anti-transglutaminase antibodies, and exclusion of gluten from the diet for at least the previous 3 months.	Unclear
(40)	Fermented sourdough wheat baked goods made from wheat flour, *Lactobacilli* and fungal proteases	Celiac disease (CD)	Proof-of-concept open challenge study	Eight patients with celiac disease aged 8–17 years	Italy
(41)	Bread	Celiac disease	Prospective cohort study	2088 Swedish Children born between 2004 and 2010 and with a genetic risk of type 1 diabetes and CD	Sweden
(42)	Fermented citrus juice	Cow milk allergy	Double-blind, randomized (1:1), two-arm, parallel-group, placebo-controlled phase 2 trial	1–18-year-old children 60 participants 30 with intervention 30 controls Median age of 5 years for both groups.	Asia
(43)	Vinegar from white wine	Anaphylaxis to egg, chicken, and lentils	Double-blind placebo-controlled food oral challenge	7 patients from 2 to 46-year-old 4 Male 3 Female	Spain
(44)	Sprouted oat fermented beverage	Celiac disease (CD)	Randomized controlled intervention study with two groups	10 adult celiac patients (22–64 years) adhering to a strict gluten-free diet for at least 2 years	Spain (conducted)
(45)	Grana Padano cheese	Egg protein allergy specifically lysosyme	Double-blind, randomized oral provocation test	Pediatric patients allergic to egg proteins, with 54 children enrolled (22 girls and 32 boys, aged 2–13 years)	Italy
(46)	Yogurt	Atopy, including conditions like atopic eczema, bronchial asthma, and food allergies	Cross-sectional study (observational study)	109 children aged 24–48 months	Ankara Turkey

**Table 3:** Outcomes of eligible studies for the systematic part.

Reference	Sample size 1*	Control	Baseline characteristics	Primary outcomes	Secondary outcomes	Side effects	PIO/PICO
(37)	Soy sauce	Soy and food constituents	All patients had negative sIgE tests for soy and wheat allergens. Positive skin prick test results to soy sauce, but not to its individual components (e.g., salt, alcohol, glucose, and amino acids).	Soy sauce allergy was confirmed through skin prick testing. The reactions were attributed to substances generated during the fermentation process.	Histamine levels in soy sauce samples were measured. Higher histamine levels were found in darker-colored soy sauces, but histamine poisoning was ruled out as a cause of symptoms.	Swelling, redness, and irritation around the lips. No systemic reactions were observed.	PICO
(38)	200 g/day of baked goods Non-fermented 8 g of gluten; Sourdough S1* 2480 ppm gluten; Sourdough S2* 8 ppm of gluten in sourdough	Non-fermented baked goods (80,127 ppm gluten)	All participants were confirmed to have normal intestinal mucosa before the study. Exclusions occurred for active serological markers or damaged duodenal mucosa at baseline.	Non-fermented baked goods caused clinical symptoms, increased antibodies (anti-tTG and EMA), and small bowel damage in all patients. Sourdough S1* baked goods caused no symptoms but induced mild mucosal inflammation in 2 patients. Sourdough S2* baked goods caused no symptoms, no antibody increases, and no intestinal mucosa changes in all participants.	Crypt proliferation and intestinal deposits of anti-tTG IgA antibodies were seen in patients consuming non-fermented or sourdough S1* baked goods. No such changes were observed in patients consuming sourdough S2* baked goods.	Non-fermented baked goods caused diarrhea, abdominal pain, and mucosal atrophy in multiple patients.	PICO
(39)	80 g of bread containing 2 g of gluten	Baker’s yeast bread fermentation	Participants were celiac patients with normal baseline intestinal permeability and negative anti-transglutaminase antibody tests.	Baker’s yeast bread caused marked alterations in intestinal permeability in 13 of 17 patients. Sourdough bread did not significantly alter intestinal permeability in the same patients.	Sourdough *Lactobacilli* extensively hydrolyzed gluten, reducing its immunotoxicity.	No adverse effect	PICO
(40)	Daily 200 g of sweet baked goods, corresponding to 100 g of processed wheat flour, which contained 10 g of hydrolyzed gluten	No positive controls.	All participants had been on a gluten-free diet for at least 3 years prior to the study, and displayed normal hematology and serology values at the study’s start.	The sourdough baked goods, containing hydrolyzed gluten, were well-tolerated. None of the participants showed changes in hematology, serology, or intestinal permeability over the 60-day trial.	Intestinal permeability (L/M ratio) remained within normal ranges (<0.03), and no clinical symptoms of gluten sensitivity were observed during the challenge.	Two participants dropped out due to difficulty adhering to the daily consumption protocol.	PIO
(41)	“Monitoring the food intake”	No control	Genetic risk of CD	High intake of bread (>18.3 g/day at 12 months) was associated with an increased risk of CD and CDA. Low porridge intake at 9 months (≤158 g/day) was associated with increased CDA risk. Milk cereal drink intake during the second year of life was linked to a higher risk of CD.	Not mentioned	No adverse	PIO
(42)	125 ml of LP0132 juice daily for 24 weeks	Citrus juice	No significant differences in demographic and clinical characteristics between groups.	The percentage of participants showing improved tolerance to cow’s milk. The results were not significantly different between groups (41.4% for LP0132 vs. 37.9% for control).	Changes in serum biomarkers, including IgE and IgG4 specific to milk proteins. Gut microbiota composition and diversity (notably increased *Lachnospiraceae* in the LP0132 group). Serum cytokine levels showed reductions in IL-5 and IL-9 in the LP0132 group.	One adverse event occurred in 10 participants (32.3%) in the LP0132 group and 8 (26.7%) in the control group, with no significant difference observed between the groups.	PICO
(43)	100 g chicken meat, 200 g lentils 5 ml Vinegar	Extract of chicken meat, egg, and lentils without vinegar	Detailed demographic and clinical data of the patients were provided, including age, sex, reported symptoms (e.g., asthma, anaphylaxis), and sIgE levels.	A 3-year-old man, lentil-sIgE of 3.08 kU/L and a wheal size of 28 mm^2^, reduced to 8.2 mm^2^ with treatment. A 46-year-old man, lentil-sIgE of 27.01 kU/L and a wheal size of 69 mm^2^, decreased to 19.9 mm^2^ with treatment.	Double-blind challenge showed tolerance to vinegar-marinated chicken, while non-marinated chicken caused urticaria	No adverse reactions	PICO
(44)	200 mL/day of SOFB	Placebo beverage made from gluten-free almond powder	No significant differences in gender, age, body mass index (BMI), or duration of gluten-free diet between groups. All participants had normal duodenal mucosa and negative IgA anti-tTG antibody levels at baseline.	SOFB consumption did not induce IgA anti-tTG antibodies or alter duodenal mucosal morphology, indicating its safety for celiac patients	SOFB consumption increased beneficial gut bacteria, such as Subdoligranulum, Ruminococcus, and Lactobacillus. A decrease in folic acid levels was noted in the SOFB group by the end of the study	No adverse effects	PICO
(45)	Every 20 min.: 0.5 g- 14.5 g. Ingested cheese was 30 g in 1 h 40 min. The amount of lysozyme was 3.63 and 3.90 mg.	Cheese without lysozyme (Trentin Grana).	Skin prick tests for lysozyme were negative in all patients. Specific serum IgE to lysozyme was used to identify sensitization (≥0.35 kU/L was considered positive).	Adverse reactions occurred in lysozyme-sensitized children when consuming Grana Padano cheese containing lysozyme. Adverse reactions were more frequent and severe with 12-month-old cheese than with 24-month-old cheese, indicating that aging reduces lysozyme allergenicity.	Severe reactions (e.g., anaphylaxis) correlated with higher lysozyme-sIgE levels (>7 kU/L).	Severe reactions include hypotension, nausea, vomiting, abdominal pain, and laryngeal angioedema. Mild reactions included urticaria, itching, and lip erythema.	PICO
(46)	Yogurt consumption is three cups per week		7% had physician-diagnosed atopic diseases (4 atopic eczema, 3 bronchial asthma, 1 food allergy).	Early introduction of cow’s milk (before 12 months) was associated with a higher risk of atopy (odds ratio: 5.59). Regular yogurt consumption showed a trend toward reducing the risk of atopy (odds ratio: 0.15, *p* = 0.08).		No side effects	PIO

1*consumed amount/frequency consumption patterns.

S1* 80 g of wheat flour and 320 g of water containing 5 x 10^8^ colony-forming units/g.

S2* 80 g of wheat flour and 320 g of water containing 5 x 10^8^ colony-forming units/g and 200 ppm of both fungal proteases.

In the study by Greco et al. (38), fermented sourdough of wheat flour was investigated to determine whether it can sufficiently reduce the gluten content in baked goods to make them safe for CD patients. Fermentation of sourdough was performed with selected lactobacilli strains and fungal proteases. For this trial, sixteen CD patients (median age, 19 years old) who were on a strict gluten-free diet were assigned to consume 200 g/day of baked goods over 60 days. The first group consumed baked goods produced with natural wheat flour with around 80,000 ppm gluten, the second group consumed partially hydrolyzed sourdough with 2,480 ppm gluten, and the last group consumed fully hydrolyzed sourdough with around 8 ppm gluten. In the first group, two patients withdrew from the study due to symptoms such as malaise, abdominal pain, and diarrhea, while all other participants developed anti-tissue transglutaminase antibodies and intestinal mucosal damage. In the second group, two patients showed subtotal villous atrophy, and one of them developed elevated antibodies. In contrast, the group with fully hydrolyzed sourdough showed no symptoms and no histological or serological changes.

Individuals in this group had no increase in intraepithelial lymphocytes and no crypt hyperplasia, and did not develop IgA-tTG deposits in the intestinal mucosa. This indicates that wheat flour is extensively hydrolyzed during sourdough fermentation to below 10 ppm gluten content may be non-allergenic for CD patients.

Current cereal baked goods are manufactured by fast processes in which long-time fermentation by sourdough has been almost completely replaced by yeast leavening agents. Under these conditions, cereal components (e.g., proteins) are not degraded during manufacture. Di Cagno et al. (39) explored the potential of sourdough fermentation to reduce gluten intolerance in celiac sprue (CS) patients. The food investigated was sourdough bread fermented for 24 h, produced using selected lactobacilli known to hydrolyze Proline-rich peptides. The type of allergy addressed was CS, an autoimmune enteropathy triggered by gluten. The study employed a combination of *in vitro* and *in vivo* methods. In *in vitro* method, lactobacilli strains were screened for peptidase activity, and a sourdough was prepared using a blend of wheat (30%) and non-toxic flours (oat, millet, buckwheat), inoculated with the selected lactobacilli. Protein hydrolysis was assessed using two-dimensional electrophoresis (2-DE) on prolamin fractions, and peptide profiles were analyzed by reverse-phase fast protein liquid chromatography (RP-FPLC). In the *in vivo* method, a double-blind acute 2-day challenge was conducted with 17 diagnosed CS patients, who consumed bread containing approximately 2 g of gluten from either baker’s yeast or the specific sourdough bread. Intestinal permeability was assessed via excreted rhamnose and lactulose levels. Results showed that sourdough fermentation nearly completely hydrolyzed wheat gliadins. In the *in vivo* challenge, 13 of 17 patients exhibited altered intestinal permeability after consuming baker’s yeast bread, whereas the same patients showed no significant changes in intestinal permeability compared to baseline after consuming the sourdough bread. The remaining four patients did not respond to gluten from either bread type. These findings support the use of sourdough fermentation with selected lactobacilli as a novel bread-making technology to decrease the level of gluten intolerance in humans. In a similar study, Di Cagno et al. (40) performed near-complete gluten degradation (<10 ppm gluten) by extending the sourdough fermentation from 24 h to 48 h and adding fungal proteases to produce sweet baked goods. They tested the goods in 8 pediatric CD patients in a chronic 60-day intervention, and no clinical, serological, or mucosal activation was observed, as assessed by RT-qPCR and ELISA assessments of IFN-*γ* expression in *ex vivo* duodenal biopsies from the patients.

Segerstad et al. (41) examined whether intake of different gluten-containing foods confers different risks of celiac disease autoimmunity (CDA) and CD in children younger than 2 years of age at genetic risk. For this purpose, the consumption of different products such as bread, baked goods, pizza, porridge, and milk cereal drink (a type of follow-on formula composed of skim milk powder and flour from different grains) was recorded through food diaries of the infants. The level of tissue transglutaminase autoantibodies was analyzed regularly for each patient until the annual screening for CD, which starts at the age of 24 months. Surprisingly, the children reported a high daily intake of bread (corresponding to a gluten intake >1 g), compared with those with no bread consumption at until 12 months of age, had almost 2-fold increased risk of developing CD. In addition, an association with increased risk of CD was also found for the children at the age of 18 months who consumed the milk cereal drink containing up to 1.5 g of gluten per bottle daily. Apart from bread and milk cereal drink, no other association was observed for other gluten sources up to the age of 24 months, and the risk of developing CDA or CD.

Yamamoto-Hanada et al. (42) conducted a 24-week, double-blind, randomized (1:1), two-arm, parallel-group, placebo-controlled, phase 2 trial study. In the intervention group, children aged 1–18 years received 125 mL of *L. plantarum* YIT 0132 (LP0132) fermented citrus juice, while the control group received citrus juice without fermentation. Of note, after fermentation, the citrus juice was pasteurized in order to heat-kill the LP0132 fermenting bacteria. Slow up-dosing cow’s milk oral immunotherapy (CM-OIT) was performed in both groups by incorporating cow’s milk amount into steamed buns. CM oral challenge was administered before starting with the intervention, which served as the baseline. After 24 weeks, effectiveness was assessed by evaluating the modulation of the Th1/Th2 balance. Among the 30 children who consumed fermented citrus juice, no significant differences were observed in total IgE, CM-sIgE, casein-sIgE, and *β*-lactoglobulin-sIgE levels compared to the control group. However, among the different antibodies produced against the various proteins in cow’s milk due to CM-OIT intervention, serum-specific β-lactoglobulin-sIgG4 titers showed a significant reduction in the LP0132 group compared with the control. Moreover, serum IL-5 and IL-9 levels were significantly lower in the LP0132 group than in the control group. The authors speculate that oral immune tolerance is promoted by LP0132 as it is taken up by macrophages in the gastrointestinal mucosa. This mechanism increased IL-10 production from macrophages and decreased Th2 cytokine production (IL-5, IL-9), thereby suppressing the Th2 responses.

Armentia et al. (43) investigated the potential of vinegar to reduce the allergenicity of egg, chicken, and lentils by combining *in vivo* and *in vitro* methods. In this study, seven patients with confirmed anaphylaxis to these foods were selected from a Spanish allergy database. Patients were diagnosed by SPT, sIgE, and double-blind oral food challenges. *In vivo* SPT with commercial and in-house allergen extracts, prepared with and without white wine vinegar, was performed on patients and controls. Only one patient underwent a double-blind, placebo-controlled food challenge with vinegar-marinated chicken. *In vitro*, IgE-immunoblots were performed on lentil and chicken extracts with and without vinegar using pooled patient sera. It was reported that vinegar, treated lentil proteins in the range of 30–90 kDa showed a reduction in IgE-binding compared with untreated ones. Furthermore, in chicken extract treated with vinegar, no IgE-binding was detected. In contrast, multiple bands were recorded in the non-treated extract. The challenged patient tolerated vinegar-marinated chicken but reacted to chicken without vinegar. These findings suggest vinegar may alter food allergen structure, promoting digestive enzyme activity, decreasing their IgE reactivity, and potentially mitigating food hypersensitivity.

In their study, Aparicio-Garcia et al. (44) examined the influence of the consumption of an SOFB in CD. The authors conducted a randomized controlled intervention study in Spain with two groups. In total, 10 adult celiac patients from 22 to 64 years who followed a strict gluten-free diet for at least 2 years prior to the initiation of the study were included. During the study, they consumed either 200 mL per day of the SOFB or a control placebo beverage made from gluten-free almond powder. Results showed that the consumption of SOFB did not induce immunoglobulin A (IgA) antibodies against tissue transglutaminase (anti-tTG) or alter duodenal mucosal morphology, indicating that it was safe for celiac patients. Moreover, SOFB consumption increased the abundance of beneficial gut bacteria, such as *Subdoligranulum*, *Ruminococcus*, and *Lactobacillus*. In addition, a decrease in folic acid levels was noted in individuals who consumed SOFB by the end of the study. No adverse effects were reported.

Lysozyme is a potential food allergen used in the dairy industry to prevent late blowing caused by the outgrowth of clostridial spores (*Clostridium butyricum* and *C. tyrobutyricum*) during cheese aging. Marseglia et al. (45) demonstrated that when lysozyme-sensitized children consumed lysozyme-containing cheese, adverse reactions could be seen in some of them, but not after the consumption of the ripened cheese. In a double-blind oral provocation study, 54 children with confirmed egg allergy were tested with Grana Padano cheese containing lysozyme and a lysozyme-free counterpart (Trentin Grana), aged 12 and 24 months. Among 21 children with detectable lysozyme-sIgE, five experienced adverse reactions, including vomiting, abdominal pain, urticaria, and one case of anaphylaxis after the consumption of 12-month-aged lysozyme-containing cheese. In contrast, only one child reacted to the 24-month-aged cheese, suggesting that extended ripening may reduce the allergenicity of lysozyme, likely due to hydrolysis of antigenic epitopes. No reactions were observed in children without lysozyme-sIgE, while some mild reactions in non-sensitized participants were attributed to high histamine levels in the aged cheese. The authors concluded that long-aged Grana Padano may be better tolerated by lysozyme-sensitized children. However, it is emphasized that caution remains necessary, especially in the case of individuals with high lysozyme-specific IgE levels, since even extended aging may not completely eliminate the risk of adverse reactions.

Özmert et al. (46), investigated factors associated with atopy in 109 children aged 24–48 months (mean age: 31.6 ± 3.5 months) in a cross-sectional study. In this study, both physician-diagnosed allergic conditions and sensitization via SPT were assessed. While 13% of children were sensitized positively based on the SPT, only 7% of children had physician-confirmed atopic diseases, 4 with eczema, 3 with asthma, and 1 with food allergy. Consuming cow’s milk before 12 months of age was found to increase the risk for atopy based on multivariate analysis. However, regular yogurt consumption over three cups per week was linked with a lower risk of developing atopy. Contrarily, having an older sibling or being exposed to maternal smoking was recorded to slightly increase the risk. All children had been breastfed, and no association was found between exclusive breastfeeding duration and atopy. The findings suggest dietary exposures and early-life environments may influence allergic sensitization in Turkish children.

In general, the outcomes from the aforementioned studies suggest that the fermentation process can modulate the allergenicity of foods, offering benefits for allergic individuals. The fermentation process with selected LAB strains has been shown to degrade allergenic proteins in wheat products and sourdough bread, making them safe for celiac patients. Likewise, treatment with vinegar from fermented wine and long ripening of cheese were shown to reduce allergic reactions in egg and lysozyme allergic individuals. Additionally, the use of *Lac. plantarum* combined with oral immunotherapy may improve the tolerance of children with cow’s milk allergy. Moreover, dietary exposure to fermented oat beverages and sourdough products has shown promising results for CD patients, even though large-scale trials are still necessary for further confirmation. Nevertheless, consumption of FFs may induce some allergic reaction, since fermentation can also generate unknown allergic compounds during the process, as evidenced by the study concerning soy sauce allergy. These results underline the importance of evaluating and characterizing the mechanism of action of the fermentation process in allergic components of food matrices and the modulation of allergic reactions for different food allergies.

### 3.4 Results of risk of bias assessment

According to the ROB 2.0 assessment of the risk of bias of the relevant studies (Table 4), only Yamamoto-Hanada et al. (42) seems to have conducted a proper randomized, parallel-group, double-blind, placebo-controlled trial. The study clearly described correct randomization and allocation concealment. Both the intervention (fermented citrus juice with heat-killed *L. plantarum*) and placebo were indistinguishable, while outcomes were blinded to the assessors. The anticipated primary and secondary outcomes were defined early in the study and guided the results presented, minimizing the risk for selective reporting. The rest of the studies assessed with ROB 2.0 appear to have a high risk of bias due to multiple reasons. The bias of the randomization process was found to be high for the Greco et al. (38) study, while those of the studies of Di Cagno et al. and Aparicio-García et al. received “some concerns” (39, 44). The Di Cagno et al. (39) study was the only crossover study of the group and had some uncertainty in Domain S, which is related to the proper management of potential carryover effects. In Domain 2, which evaluates the risk of bias due to deviations from the intended interventions, only the Yamamoto-Hanada et al. (42) study was assessed using the “effect of assignment to intervention” approach due to its proper randomization. The other three studies were evaluated with the “effect of adhering to intervention” approach since their randomization was problematic or uncertain. In this domain, deviations from the intended intervention, including the monitoring of the adherence of participants, were assessed as high risk for both the Greco et al. (38) and the Di Cagno et al. (39) studies, while the study by Aparicio-García et al. (44) was evaluated with “some concerns.” Domains 3, 4, and 5 concerning bias due to missing outcome data, bias in measured outcomes, and bias due to selective reporting, respectively, were evaluated in most cases as low risk, with a few exceptions that received “some concerns.” As mentioned above, all these uncertainties resulted in all studies being evaluated having a high risk of bias overall, and only the Yamamoto-Hanada et al. (42) study was evaluated as having a low risk of bias.

**Table 4:** Quality assessment of studies based on ROB 2.0.

Study	Type of RCT parallel (P) or cross-over (CO)	Domain 1a: Risk of bias arising from the randomization process	Domain S: Risk of bias arising from period and carryover effects (Only for cross-over)	Domain 2: Risk of bias due to deviations from the intended interventions (effect of assignment to intervention)	Domain 2: Risk of bias due to deviations from the intended interventions (effect of adhering to the intervention)	Domain 3: Risk of bias due to missing outcome data	Domain 4: Risk of bias in measurement of the outcome	Domain 5: Risk of bias in the selection of the reported result	Overall risk of bias
(38)	P	High	NA	NA	High	Some concerns	Low	Some concerns	High
(39)	CO	Some concerns	Some concerns	NA	High	Low	Low	Some concerns	High
(42)	P	Low	NA	Low	NA	Low	Low	Low	Low
(44)	P	Some concerns	NA	NA	Some concerns	Low	Some concerns	Some concerns	High

Furthermore, four studies were assessed by ROBINS-I as they were identified as interventions that were not randomized (Table 5). The study by Marseglia et al. (45) is a double-blind crossover intervention, the study by Sugiura and Sugiura (37) is a case series based on clinical observations and prick tests, the study by Di Cagno et al. (40) is a pilot intervention study, while the study by Armentia et al. (43) is an exploratory, non-randomized interventional study. During the assessment, all four studies resulted in a serious or a critical overall judgment of risk of bias. For Domain 1, confounding was found to be serious for Marseglia et al. (45), Di Cagno et al. (40), and Armentia et al. (43) or critical for Sugiura and Sugiura (37). These evaluations can be assigned when there is a lack of a comparator or control group, a high potential for uncontrolled confounding variables, and/or a lack of adjustment for baseline differences. In Domain 2, Sugiura and Sugiura (37) were rated as critical for bias in the classification of interventions, as there was no standardized exposure protocol described, and the allergic responses were attributed to complex soy sauce components. The other three studies received low and moderate risk of bias. In Domain 3, all four studies did relatively well, receiving low to moderate judgments, suggesting that the eligibility criteria for the selection of participants were clearly defined, and appropriate enrollment procedures were applied. In Domains 4, 5, 6, and 7, Marseglia et al. (45) and Di Cagno et al. (40) were also rated mostly with low or moderate risk of bias. This shows that deviations from intended interventions, missing data, measurement of the outcome, and selection of the reported result did not lead to any major risk of bias, and they were generally well-managed, having a limited impact on the validity of the findings. However, the other two studies faced a serious risk of bias in Domain 7. In the Sugiura and Sugiura (37) study, the results reported seemed to be rather selective and descriptive, which may have resulted in the overestimation of the clinical significance of soy sauce as an allergen. In the study of Armentia et al. (43), there was a lack of a pre-registered protocol or analysis plan, which could lead to selective reporting of favorable outcomes.

**Table 5:** Quality assessment of studies based on ROBIN I.

Study	Study type	Domain 1: Bias due to confounding	Domain 2: Bias in classification of interventions	Domain 3: Bias in selection of participants into the study (or analysis)	Domain 4: Bias due to deviations from intended interventions	Domain 5: Bias due to missing data	Domain 6: Bias in measurement of the outcome	Domain 7: Bias in selection of the reported result	Overall Judgment
(45)	Double-blind non-randomized crossover trial	Serious	Low	Low	Low	Low	Low	Moderate	Serious
(37)	Case series with diagnostic testing	Critical	Critical	Moderate	Moderate	Low	Moderate	Serious	Critical
(40)	Non-randomized, single-arm intervention (pilot study)	Serious	Moderate	Moderate	Moderate	Moderate	Moderate	Moderate	Serious
(43)	Exploratory, non-randomized interventional study	Serious	Moderate	Low	Low	Moderate	Moderate	Serious	Serious

The final two studies by Segerstad et al. (41) and Özmert et al. (46) were assessed by the NOS risk of bias tool as they were identified as cohort and cross-sectional observational studies (Table 6), respectively. The Segerstad et al. (41) study was found to be of high quality since it received top scores for all NOS categories. It involved a large, multicenter sample size, controlled multiple important confounders, and outcomes were defined objectively using serologic markers and biopsies. In addition, there was a detailed follow-up for up to 10 years. The Özmert et al. (46) study was found to be of good/borderline quality, suggesting some hindering factors in the overall design. Selection was likely biased since the study was conducted with a convenience sample of children, limiting randomness and reproducibility. In addition, exposure classification was retrospective and relied on self-reported behaviors. The study was adjusted for several potential confounders, but the outcomes were subjective and lacked external validation.

**Table 6:** Quality assessment of studies based on NOS.

Study	Study type	Selection (max 4*)	Comparability (max 2*)	Outcome/Exposure (max 3*)	Total score (max 9*)	Overall risk of bias
(41)	Cohort	4	2	3	9	High quality
(46)	Cross-sectional	2	2	2	6	Good quality (borderline)

### 3.5 Mechanism of action proposed in the human studies analyzed in the systematic review

The first suggested mechanism concerns the degradation of allergenic food proteins mediated during fermentation. Three articles reported results from clinical challenges in patients with CD or gluten-sensitive enteropathy (38–40). The challenges involved the consumption of bread and baked goods made with wheat flour fermented by a specific pool of *Lactobacillus* strains. The Lactobacillus strains involved in fermentation exhibited/expressed proteolytic activity targeting gluten proteins rich in proline and glutamine, resulting in nearly complete degradation of gluten and gliadin within 24 h. *Lactobacillus* led to pre-digestion of the toxic 33-mer peptides from gliadin, preventing their presence in the small intestine. In these studies, fungal proteases from *A. oryzae* and *A. niger* or cytoplasmic extracts were added to further enhance the proteolytic activity of the lactobacilli. The studies demonstrated a complete degradation of gluten, including its prolamin components. Furthermore, they confirmed safety by showing no signs of inflammation or intestinal permeability impairment. Moreover, one study showed that SOFB with specific *Lactobacillus* strains effectively degraded immunogenic prolamin peptides, making it safe for celiac patients. The mechanism is based on proteolysis and degradation of components that stimulate interferon-*γ* (IFN-γ), hence losing the immunogenicity (44).

Furthermore, another paper highlights the role of vinegar to improve food digestion and consequently, to reduce the risk of food allergies (due to egg, chicken, and lentils) (43). This FF acts by increasing gastric acidity and facilitating the breakdown of food proteins, mimicking the natural gastric digestion. This process minimizes the risk of allergic reactions, mitigating the allergic responses in sensitized individuals, especially in IgE-mediated food allergies.

In addition, an oral provocation test, with varying amounts of cheese (with and without lysozyme), showed that cheese aging may reduce the severity of allergic reactions to egg lysozyme, particularly when it is extended over 12 months (45). This may be due to a modification of the allergenic epitopes of the protein. Prolonged ripening may alter lysozyme structure and reduce its IgE-binding potential, this way, it will be less reactive, as suggested by the authors of the study.

Consumption of the FFs may also relate to favorable changes of the gut microbiome and promote antiallergenic processes. Accordingly, SOFB administration in CD patients led to an increase in beneficial bacteria, including *Subdoligranulum*, *Ruminococcus*, and *Lactobacillus*. This shift in microbiota composition could be associated with improved immune responses and reduced production of pro-inflammatory cytokines (44). The study by Özmert et al. (46) also suggests that regular consumption of yogurt supports the health gut microbiome plays a critical role in immune system development and oral tolerance to food antigens (46). Probiotic modulation of gut microbiota can be combined with treatments such as oral immunotherapy (OIT), which has arisen as a promising approach for food allergies (e.g., cow’s milk, peanut), aiming at inducing tolerance. For example, heat-killed LP0132 with OIT was effective for alleviating IgE-mediated cow milk allergy (42). Heat-killed LP0132 induced IL-10, a key anti-inflammatory cytokine which regulates Th2 responses and mediates allergic reactions.

While fermentation can be a beneficial process for reducing allergenicity, this is not definitive, since even if allergens are not detected in the FFs, the fermentation could lead to the generation of compounds with potential allergic capacity. Sugiura & Sugiura (37) hypothesized that the soy sauce allergy in their patients could be caused by the substances produced during fermentation that were different from histamine. However, the specific allergenic agents were not identified.

### 3.6 Mechanism of action supported by animal and *in vitro* studies

The effects and roles of many different FF groups on allergic responses (AR) for alleviation or aggravation are still controversial. Normally, some of the aforementioned matrices in a non-fermented condition aggravate AR by stimulating other immune cells (e.g., mast cells and other granulocytes), which altogether attack the allergen and cause AR symptoms (e.g., dermatitis, rhinitis, asthma, eczema). Recently, many *in vitro* and *in vivo* studies were undertaken to support human studies explaining how fermentation can help restore the mechanism based on the imbalance of T cells subtypes 1 and 2 (Th1/Th2 switch), modulating the release of Th2-pro interleukin (IL), chemokines, and antibodies such as IgE antibodies by B cells. However, supportive evidence is still necessary to understand the health-promoting aspect of FFs. Recent studies are presented herein that support the consumption of FFs, used for the systematic part of this review, which have also shown a potential for the mitigation of AR, both *in vitro* and *in vivo*.

Indeed, it is well established that FFs can act at different levels, by (1) enhancing the proteolytic degradation of allergenic proteins before gastrointestinal digestion, (2) inhibiting the stimulatory activity on T-cells, or (3) modulating the host immune response. For instance, fermentation utilizes proteolytic enzymes from LAB and/or fungi to degrade allergenic proteins, such as proline-rich peptides found in gliadin (e.g., the 33-mer peptide in celiac disease) and other allergens. This process is essential in products like sourdough bread, oat beverages, Grana Padano cheese, and soybean meal. LAB, like *Lacticaseibacillus casei*, has been shown to metabolize the 33-mer peptide from gliadin, reducing its immunogenicity (47). Similarly, the use of LAB and fungal proteases in sourdough fermentation effectively degrades gluten proteins, including gliadins, into smaller, less immunogenic peptides (48). In dairy products such as Grana Padano cheese, the immunoregulatory effect on food allergy arises from peptides produced during fermentation; the higher ripening time is strictly correlated to a massive protein hydrolysis and consequently to possible strong tolerogenic effects in allergic patients (49). In addition, in soybean meal, a solid-state fermentation is realized through a mixture of *Lct. casei*, yeast, and *Bacillus subtilis*, exhibited a lower *in vitro* IgE-binding capacity, as measured by the competitive inhibition ELISA, than that of the non-fermented soybean meal (50).

Another key beneficial aspect of FFs in food allergy is the inhibition of stimulatory activity on T-cells. Allergens are often composed of proteins and are inert, but are recognized as antigens or ‘invaders’ by the immune system. This recognition causes an imbalance of T cell subtypes 1 and 2 (Th1/Th2 switch), resulting in the release of Th2-pro interleukin, chemokines, and antibodies such as IgE antibodies by B cells. The protein hydrolysis carried out from the fermentation processes can reduce the imbalance effects on the immune system, for example, acting on the non-stimulation of IFN-*γ* or IL-2 production in CD4 + T-cells in sensitized individuals, which normally occurs after the ingestion of intact allergen, or involving loss of HLA-DQ2/DQ8 binding capacity in individuals with CD. In this context, Levescot et al. (51) showed that the incomplete digestion of gluten prolamins in the intestinal lumen triggers a toxic immune response. This involves the binding of deamidated peptides, rich in proline and glutamine, to HLA-DQ2 or HLA-DQ8 molecules on antigen-presenting cells (APCs), leading to the activation of CD4 + T cells. These T cells release proinflammatory cytokines, IL-2, IFN*γ*, and IL-21, which contribute to intestinal inflammation and tissue damage (51). In addition, the elimination of immunogenic gluten epitopes can prevent this inflammatory cascade and protect the intestinal epithelium. This has been demonstrated through the fermentation of wheat flour (52) and durum wheat semolina (53), using sourdough lactobacilli and fungal proteases. The fermentation process fragmented the immunogenic epitopes, rendering them incapable of stimulating immune responses in cells derived from CD patients, including PBMCs, intestinal T cells, and organ cultures from intestinal biopsies. As a result, levels of IFN-γ and IL-2 remained comparable to those in untreated control cells (52, 53). Likewise, Lee et al. (54) reported that consumption of Cheonggukjang (CKJ), a fermented soybean product, can significantly reduce allergic reactions in transgenic mice, decreasing luciferase signals, dermis thickness, auricular lymph node weight, and mast cell infiltration. Although serum IgE levels remained unchanged, CKJ lowered pro-inflammatory cytokines IL-6 and VEGF expression. Similarly, to Lee et al. (54), a study conducted on the BALB/c model using a fermented soybean meal indicated that the group fed with fermented soybean meal manifested milder damage to the intestine compared to the control group, with lower mMCP-1 and IgE levels.

The effects of fermentation on the reduction of the immune system imbalance do not solely arise from protein hydrolysis but could potentially be ascribed to pH changes that occur in the food matrix during the fermentation process. For example, it was demonstrated that vinegar can have an impact on food matrices, reducing the matrix pH and inducing conformational changes in protein structures (55). Magalhães et al. (56) reported that adding vinegar (4%) to the dough during bread baking caused a significant immunogenic reduction of gliadin by about 44% at the end of the intestinal digestion phase, compared to the control bread containing gluten, suggesting that the addition of vinegar may help in the hydrolysis of immunogenic gliadin sequences at the end of the digestion process. Nevertheless, through the use of low-pH incubation, it is also possible to treat lectin allergenicity, obtaining a weaker anaphylaxis response, as well as a significant reduction of the release of IgE, IgG1, histamine, mMCPT-1, and cytokines in BALB/c mice (57).

The modulation of immune response in allergic patients can also be achieved by the addition of an exogenous source of beneficial microbes, like probiotic bacteria, which can directly modulate macrophage functionality in a strain-specific and subset-dependent manner, protecting the epithelial barrier and affecting pro-inflammatory and anti-inflammatory cytokine secretion. Foey et al. (58) reported that in a co-culture model of Caco-2 cells with macrophages, *Lct. casei* Shirota rescues epithelial barrier integrity (ZO-1 expression and TEER) and modulates cytokine secretion in an inflammatory condition. Moreover, *Lct. casei* Shirota attenuated the immune responses against OVA by reducing the proliferation of splenocytes, levels of OVA-specific IgE, immunoglobulin G (IgG), and IgM, and ratio of Th2/Th1 cytokines (59). Similarly, LP0132 derives from FFs, has been found to induce a high level of IL-10 secretion from murine macrophages (60).

Moreover, in recent years, significant advances have been made to understand how live starter cultures and their fermentation-derived metabolites, such as short-chain fatty acids (SCFA), bioactive peptides, and exopolysaccharides can act synergistically to enhance microbial diversity, reinforce epithelial barrier integrity via upregulation of tight-junction proteins, and modulate immune signaling, offering the potential to treat food allergies and induce intolerance (61). Among fermentation-derived metabolites, SCFAs generated during dairy fermentation seem to exert precise effects on gut microbial communities and host physiology, regulating the immune system and preventing excessive inflammation. Fermented milk (FM) has an inhibitory effect on stress-induced visceral hypersensitivity by preventing stress-induced increases in intestinal permeability and restoring tight junction protein expression to control levels (62). Moreover, kefir supplementation elevated levels of proximal colonic SCFAs, upregulated tight-junction proteins (e.g., ZO-1), and decreased systemic lipopolysaccharide (LPS) levels in rodent colitis models (63). Furthermore, combining butyrate-enriched fermented milk reduced colonic nuclear factor kappa-light-chain-enhancer of activated B cells (NF-κB) activation in murine models, highlighting synergistic anti-inflammatory effects (64). These synergistic effects due to the mitigation of inflammatory reaction and changes in microbial metabolites have been seen, also in a matrix different from dairy, like a sprouted oat fermented beverage (44). As already mentioned, Aparicio-García et al. (44) had developed a novel beverage from sprouted oat flour by fermentation with *Lac. plantarum*. This beverage showed an *in vitro* reactivity against anti-gliadin antibodies (AGA) and anti-inflammatory potential in RAW 264.7 macrophages and Caco-2 cells. In addition, it changed the gut microbiota composition, leading to a higher relative abundance of some beneficial bacteria, including the genera *Subdoligranulum*, *Ruminococcus,* and *Lactobacillus*.

Furthermore, the use of fermentation in fruit matrices, as mango, has shown promising *in vitro* results. Tian et al. (65) demonstrated that juice fermentation of mango fruits with 15% kombucha starter culture reduces IgE reactivity by ELISA test with sera of allergic patients. Like mango, another fermented multi-fruit beverage, made from five fruits and fermented by *Saccharomyces cerevisiae,* along with LAB and acetic acid bacteria, demonstrated immunomodulatory properties *in vivo*, both in non-specific and ovalbumen (OVA)-specific immune response experiments using female BALB/c mice (66). Administration of the fermented multi-fruit beverage did not affect B cell proliferation and IgG production. In addition, the analysis of cytokine secretion profile also revealed that the fermented multi-fruit beverage decreased the production of proinflammatory cytokines IL-6 and TNF-*α* production in OVA-immunized mice. It also caused a decrease in the production of anti-OVA IgG1, which was accompanied by a decrease in Th2-related cytokines IL-4 and IL-5 production and an increase in Th1-related cytokine IFN-*γ* production, indicating that it may have the potential to shift the immune system from the allergen-specific Th2 responses toward Th1-type responses.

## 4 Conclusion

Previous studies and reviews have primarily focused on probiotic supplementation or isolated fermented matrices, often overlooking the broader spectrum of fermented foods consumed as part of the daily diet. In contrast, the present systematic review provides a comprehensive synthesis of the available human studies assessing the impact of fermented food consumption on food allergies, systematically mapping the evidence across diverse food categories, evaluating study quality using design-specific risk of bias tools, and integrating the findings within a mechanistic and clinical context. Based on the string used to search the literature databases, relatively few papers met the relevant criteria. An evaluation of these studies indicates that fermentation may reduce the allergenicity of specific food components, especially food proteins (e.g., gluten and soy proteins), presumably through substrate modification or degradation. However, the effects observed seem to be dependent on the specific food matrix, the fermenting microbial strains employed, or production parameters like the duration of ripening. Nevertheless, fermentation may also be ineffective or even increase allergenic potential as in the study of fermented soy described earlier. Although several studies reported reduced allergenicity due to fermentation-mediated hydrolysis of allergenic proteins, it is equally important to acknowledge that in at least one case, fermentation was associated with an aggravation of allergic responses, highlighting that its effects are not uniformly beneficial. Therefore, the interpretation of current evidence should remain cautious, as the available human studies are limited in number, heterogeneous in design, and do not yet allow firm conclusions on the safety or efficacy of fermented foods for allergic individuals. The literature supports an alternative pathway beyond allergen degradation, which is the direct immune modulation of the host by fermenting food. This can take place either through the direct interaction of the fermenting microorganisms and the host’s immune system or through the regulation of the host’s gut microbiota by specific food constituents (e.g., prebiotics).

Although these findings are promising, the current body of evidence remains preliminary, with most studies being small-scale, underpowered, and methodologically limited. In addition, these limitations prevent any conclusions regarding the role of FFs in food allergies from being reliably applied or considered by regulatory authorities. To advance the field, future clinical trials should adopt standardized definitions of fermented foods, harmonized allergenicity outcome measures, and include long-term follow-up to assess both safety and sustained effects. In detail, based on the risk of bias assessment, several studies analyzed here suffered from methodological concerns, including small sample sizes, heterogeneous study designs, limited allergen diversity, short duration, and lack of long-term follow-up, unstandardized fermentation protocols, and limited biomarker analysis. High-quality, multicenter randomized controlled trials (RCTs) should be conducted that integrate robust randomization, adequate blinding, and predefined endpoints. It is also necessary to expand both the allergen targets and the age groups while developing standardized FFs interventions that are necessary for establishing reproducibility among the studies, and perhaps most importantly, allowing the identification of the mechanistic insight for the mode of action. This last point is very important since it will form the basis for rationally assessing the effect of FFs on food allergies, which will allow further improvements in the future. This could also be supported by the integration of high-throughput multi-omics for both the analysis of FFs and the extensive immune profiling of the host. Moreover, considering individual sensitivities and detailed stratification of the subjects will allow deeper insight and will reveal applications beyond specific populations. It will also be vital to evaluate the long-term safety and efficacy of FFs, given that currently we rely solely on their safe history of consumption. It is plausible to hypothesize that, given enough state-of-the-art studies, it may be feasible to develop regulatory and clinical guidelines about the application of fermented foods in clinical nutrition as supplementary or even first-line defenses against food allergies.

## Data Availability

The original contributions presented in the study are included in the article/Supplementary material, further inquiries can be directed to the corresponding authors.

## Footnotes

1
http://www.ohri.ca/programs/clinical_epidemiology/oxford.asp
